# DNMT1 Gene Expression in Patients with *Helicobacter pylori* Infection

**DOI:** 10.1155/2022/2386891

**Published:** 2022-09-13

**Authors:** Manouchehr Ahmadi Hedayati, Amjad Ahmadi, Zahed Khatooni

**Affiliations:** ^1^Liver and Digestive Research Center, Research Institute for Health Development, Kurdistan University of Medical Sciences, Sanandaj, Iran; ^2^Department of Microbiology, Faculty of Medicine, Kurdistan University of Medical Sciences, Sanandaj, Iran; ^3^Department of Microbiology, School of Medicine, Hamadan University of Medical Sciences, Hamadan, Iran; ^4^Cellular and Molecular Research Center, Research Institute for Health Development, Kurdistan University of Medical Sciences, Sanandaj, Iran; ^5^School of Pharmacy, Memorial University of Newfoundland, St Johns, NL, Canada

## Abstract

DNMT1, as a critical enzyme affecting epigenetics through methylation of DNA cytosine-rich sequences, regulates gene expression. Exterior factors including long-term infections, in this study *Helicobacter pylori* infection, could change host cells' epigenetics by affecting *DNMT1* gene expression. This study investigated the statistical correlation between *H. pylori* virulence genes and *DNMT1* gene expression in gastric antral epithelial cells of gastric adenocarcinoma and gastritis patients. In a case-control study, 50 and 53 gastritis and gastric adenocarcinoma antral biopsies, including 23 and 21 patients with *H. pylori* infection, respectively, were collected from hospitals in the west of Iran. Having extracted total RNA from gastric biopsy samples, cDNA was synthesized and virulence genes of *H. pylori* were detected by using the PCR method. Relative real-time RT PCR was used to detect ΔΔCt fold changes of the *DNMT1* gene expression in divided groups of patients based on *H. pylori* infection and clinical manifestations. The results showed that along with increasing patients' age, the *DNMT1* gene expression will increase in gastric antral epithelial cells of gastric cancer patients (*P* ≤ 0.05). On the other hand, the biopsy samples with infection of *H. pylori cagA*, *cagY*, and *cagE* genotypes revealed a direct correlation along with increased *DNMT1* gene expression. This study revealed the correlations of *H. pylori cag* pathogenicity island genes with increased DNMT1 gene expression.

## 1. Introduction

Methylation is a common epigenetic trend in handling cell gene expression [1]. DNMT [DNA (cytosine-5)-methyltransferase] enzymes contribute to the expression of growth-promoting genes and cell differentiation by directly adding and removing methyl groups to cytosine and histones [2]. Mammalian cells' DNMTs include DNMT1, DNMT3A, and DNMT3B; in the meantime, DNMT1 methylates CpG cytosine-rich sequences in the gene's promotors of the newly synthesized DNA strand [1, 3]. The results of related studies show that functionally defective DNMT enzymes increase the incidence of adenocarcinoma [4–6]. By means of this, cell proliferation will be increased following the effects of DNMTs inactivating adenocarcinoma suppressor genes through over-methylation [1, 7]. The molecular mechanism of carcinogens is conducted through their effects on DNMT activity; for instance, microbes such as uropathogenic *E. coli* increase the *DNMT1* gene expression in host cells [8–12].

Cell signaling studies show that the APC (Adenomatous Polyposis Coli) as a tumor suppressor gene and a regulator of the Wnt signaling pathway through *β*-catenin stability and/or degradation controls *DNMT1* gene expression [13]. The Wnt (Wingless oncogene INT1) receptors affect the APC/*β*-Catenin signaling complex, then releasing *β*-Catenin and activating the TCF (T-cell factor) transcription factor following attach to the *DNMT1* gene promoter block gene expression [13–15]. In this regard, clinical complications are impressed with the direct interaction of DNMT1 protein with the APC/*β*-Catenin signaling complex that has strongly been reported in colorectal adenocarcinoma mice [15]. In addition, the basic studies show bacteria reduce the expression of Wnt/*β*-Catenin repressor genes causing an increased expression of the *DNMT1* gene [16–18]. Upregulation of Cadherin-1 (CDH1), as an inhibitor of lung cell metastasis, reduces Wnt/*β*-Catenin activity in non-small-cell lung adenocarcinoma cells [19–21]. Furthermore, methylation of the CpG sequences in the promoter of the CDH1 gene by DNMT1 leads to preventing its expression [21]. Regarding the results of molecular-oriented studies, *Helicobacter pylori* CagA (Cytotoxin-associated gene A) protein disrupts the Wnt/*β*-Catenin signaling pathway leading to an increase in *DNMT1* gene expression [22–24].

Epstein–Barr virus (EBV) as a well-known carcinogenic viral agent infects B lymphocytes and increases the activity of the *DNMT1* enzyme [25, 26]. The relevant studies show that EBV infections have a high prevalence in human societies; in addition, a small percentage of people show *H. pylori* and EBV co-infection [27–30]. This hypothesis supported that the *H. pylori* and EBV co-infection could lead to further *DNMT1* gene upregulation and accelerates the process of gastric epithelial cell carcinoma [30, 31].

This study aimed to investigate the correlation between *H. pylori* virulence genes and the *DNMT1* gene expression in gastric epithelial cells of patients.

## 2. Materials and Methods

### 2.1. Sampling

A case-control study was designed involving gastritis and gastric adenocarcinoma patients as the control and case groups, respectively. 53 and 50 patients with gastric adenocarcinoma and gastritis were enrolled. The cDNA of *H. pylori* virulence genes in biopsy specimens was detected by conventional PCR (polymerase chain reaction) [32]. The consecutive sampling method was used to collect gastric biopsy samples from patients with gastrointestinal symptoms referred to Tohid and Shahid Ghazi hospitals in Sanandaj city, located in the west of Iran [33–35]. The urea breath test was used to determine the *H. pylori* active infection [33]. Gastric antral biopsy specimens were obtained from each patient for molecular analysis of *H. pylori* genotypes, EBV infection, and *DNMT1* gene expression. The gastric biopsy specimen was immediately dropped into RNALater (Roche Co.) solution and transferred to the Molecular Microbiology Laboratory of Kurdistan University of Medical Sciences. A clinicopathological biopsy sample was collected from the gastric carcinoma tumor area of patients with gastric adenocarcinoma and transferred to Tohid Hospital Pathology Laboratory. To evaluate the clinicopathological biopsy, the Kaplan–Meier method was used, including stage (TNM system (tumor-node-metastasis)) and tumor grade (including 1, 2, 3, and 4 grades) as macroscopic and microscopic examination methods, respectively [36].

### 2.2. *H. pylori* Virulence Gene PCR

Total RNA was extracted from patients' gastric biopsy specimens by using an RNA extraction kit (Takara Co.) and immediately converted to cDNA. The extracted RNA integrity was evaluated by observing the 28S and 18S rRNA bands on the 1.2% agarose gel [37]. The absorbance ratio at 260 nm and 280 nm was assessed for the purity of RNA by using NanoDrop® 2000 spectrophotometer machine (Thermo Fisher Company, Germany) [37]. Using online primer three software (version 0.4.0), specific primers of *H. pylori* virulence genes were designed and synthesized (Bioneer, South Korea Co.). First, the *H. pylori 16s rDNA* gene primer was used to determine the *H. pylori* genome's cDNA by PCR (Table 1). Other primers of *H. pylori* virulence genes were used in the biopsy specimens of patients with positive-*H. pylori 16s rDNA* gene. The annealing temperature of *H. pylori* virulence genes' specific primers is shown in Tables 1 and 2. PCR master mix was included buffer 10X (2.5 microlitre), DNA Taq polymerase 5U/microliter (0.25 microlitre), dNTPs 10 mM (0.5 microlitre), MgCl2 50 mM (1 microlitre), cDNA (2 microlitre), forward and reverse specific primers 10 picoliter (each one 0.5 microlitre) and RNase-free water (12.75 microlitre) in final volume 20 microlitre [37]. The Thermal cycling PCR steps involved an initial denaturation at 94°C for 5 minutes, a denaturation at 94°C for 30 seconds, a primer annealing for 45 seconds (temperatures are shown in Table 1), an extension at 72°C for 45 seconds, and a final extension at 72°C for 5 minutes [37]. The PCR product was rounded on 1.5% agarose gel.

### 2.3. EBER PCR

To detect the EBV virus genome, EBER (Epstein–Barr virus-encoded small RNAs) gene's cDNA was detected by PCR in the gastric epithelial antral biopsy samples. EBER-specific primers were checked by using the Epstein–Barr virus (Human gamma herpes virus 4) gene sequence in the GenBank and online primer software 3.  Table 3 shows the sequence and annealing temperature of the EBER-specific primers. According to the standard PCR protocol, PCR was performed to determine gastric epithelial cells' infection to the EBER sequence.

### 2.4. *DNMT1* Gene Real-Time PCR

The *DNMT1*-specific primers as the target gene and GAPDH (glyceraldehyde-3-phosphate dehydrogenase) as internal genes were checked using gene sequence in the GenBank and online primer software 3 (Table 3). The PCR amplification program began with a 10 minute denaturation step at 95°C and then 40 cycles of denaturation at 95°C for 15 seconds, annealing at 60°C for 30 seconds, and extension at 72°C for 60 seconds [37]. The relative amounts of the *DNMT1* expression gene between gastritis (control group) and gastric adenocarcinoma (case group) patients were evaluated by ΔCt (threshold cycle) = Ct (*DNMT1*) Ct (GAPDH). Finally, the *DNMT1* gene expression fold changes were calculated by the 2^−ΔCt^ formula [42].

### 2.5. Statistical Methods

Statistical analysis of the results was performed using SPSS (Statistical Package for the Social Sciences) software version 26. Correlations between qualitative variables, including sex, disease, and *H. pylori* infection, were calculated using chi-square and crosstab statistical tests [35]. To investigate the *DNMT1* gene ΔCt as a quantitative variable in different groups and subgroups, the distribution of the *DNMT1* gene ΔCt was detected using the Kolmogorov–Smirnov test [35]. The *DNMT1* gene ΔCt values showed normal distribution, so Student's *T*-tests and ANOVA analysis were used to investigate the correlation between *DNMT1* gene expression in different groups [35]. A *P* value less than 0.05 was considered a statistically significant level for measures [35].

## 3. Results

The demographic results showed that gastric adenocarcinoma's prevalence gradually increased with increasing age (Table 4). The prevalence of gastric adenocarcinoma in men was 2.31-fold higher than in women (Table 4). As adenocarcinoma prevalence, the *H. pylori* infection increased with age increasing (Table 5). The highest *H. pylori* infection rate was observed in the range between 61 and 85 years old (Table 5). The prevalence of *H. pylori* infection in men in the study was 1.75-fold higher than in women (Table 5). The statistical relationship between *DNMT1* gene expression and demographic variables was investigated using the *T*-test. This study showed a gradually increased *DNMT1* gene expression with along increasing age (Table 6). In gastric antral epithelial cells of both groups of patients with *H. pylori* infection and gastric adenocarcinoma, the DNMT1 gene expression was significantly increased (Table 6). The *DNMT1* gene expression in women was 2.07-fold higher than in men, without statistical correlations (*P* ≤ 0.094) (Table 6).

A survey of cDNA of *H. pylori* virulence genes in gastric biopsy specimens revealed the lowest and highest frequencies for *cagE* and *oipA* (Outer Inflammatory Protein) in gastritis patients and *sabAB* (Sialic Acid Binding Adhesin) (the gastric biopsy samples with both *H. pylori sabA* and *sabB* genotypes infection), *oipA*, and cagT in gastric adenocarcinoma patients, respectively (Table 7). A significant difference between cDNAs ΔCt of *cag* genes was observed in gastritis biopsy specimens of gastritis patients (*P*  ≤ 0.05) (Table 7). The *H. pylori cagE*^−^, *cagA*^−^*EPIYAC*^−^, and *cagA*^−^ genotypes showed a strong correlation with gastritis (Table 7). The results of this study showed that gastric biopsy specimens with *H. pylori cagY*^*+*^ and *cagT*^*+*^ genotypes infection correlated with gastric adenocarcinoma (Table 7). The *hopQ*^−^ (*Helicobacter* Outer Membrane Protein) and *hopQII*^*-*^ genotypes of *H. pylori* were correlated with gastritis and gastric carcinoma, respectively (*P* ≤ 0.05). Gastric biopsy specimens of patients with the *H. pylori cagY*^+^, *cagE*^*+*^, and *cagA* ^*+*^ genotypes infection showed a negative correlation with the *DNMT1* gene ΔCt (Table 8). These results indicate an increase in *DNMT1* gene expression in gastric biopsy specimens (Table 9). The *DNMT1* gene expression with statistically significant differences in patients' subgroups was calculated using the ΔΔCt formula (Table 9). The results showed the lowest and highest expression of the *DNMT1* gene in the youngest and oldest patients with a range age of 30–18 and 61–85 years, respectively (Table 9). The *DNMT1* expression genes in the age group of 61–85 years were 5.32-fold higher than in 18–30 years old (Table 9). The *DNMT1* gene expression in gastric adenocarcinoma patients was 3.25-fold higher than in gastritis patients (Table 9). The difference was 3.11-fold higher in the group of patients with *H. pylori* infection than without *H. pylori* infection (Table 9). The *DNMT1* gene expression in the groups of patients with *H. pylori cagE*^*+*^, *cagY*^*+*^, and *cagA* ^*+*^ genotypes infection compared to patients with *H. pylori cagE*^−^*, cagY*^−^*, and cagA*^−^ genotypes infection was 4.09-, 3.08-, and 3.04-fold, respectively (Table 9) (*P* ≤ 0.05).

## 4. Discussion

The patients' demographic results of the current study complied with related previous studies' results [33, 43–45]. The prevalence of gastric adenocarcinoma among men was 4 times higher than among women, and the highest adenocarcinoma growth rate was observed among the oldest patients' group (Table 4). According to other study results, *H. pylori* infection is directly associated with the prevalence of gastric adenocarcinoma, however, our study results showed no significant correlation [44, 45]. In this regard, in the sampling step, we excluded patients with a history of arbitrary use of the anti-*H. pylori* chemotherapy without a medical prescription. So, it could be the possible reason for no significant correlation between *H. pylori* infection and gastric adenocarcinoma prevalence in our patients.

The DNMT1 enzyme contributes to cell proliferation by methylation and blocking the tumor suppressor genes֝ expression [1]. The *DNMT1* gene֝s expression is a consequence of cell signaling pathways leading to cell growth and proliferation [1]. According to the results of previous studies, the *DNMT1* gene's expression in gastric epithelial cells increases with age increasing, gastric adenocarcinoma, and *H. pylori cagA* genotype infection [46–51]. Previous studies show that the *H. pylori* CagA oncoprotein interferes with the Wnt/*β*-Catenin signaling pathway and increases *DNMT1* gene expression and cell proliferation [22, 23]. CagA inhibits GSK3 (Glycogen Synthase Kinase 3) activity by stimulating c-Met via the PI3K-AKT pathway [22]. By inactivating GSK3, the *β*-Catenin/TCF complex is inactivated, and the *DNMT1* gene is expressed [13, 15, 16, 18]. The *H. pylori* VacA inhibits GSK3 activity by influencing EGFR receptors via the PI3K-AKT pathway [22, 52, 53]. Some studies show that *H. pylori* CagA proteins also increase the DNMT1 gene expression and accelerate the methylation process of tumor suppressor genes by affecting integrin receptors and the JAK/STAT3 signaling pathway [22]. The results of the current study were consistent with the Ma et al. (2017) study that showed an increased *DNMT1* gene expression increases the chance of developing gastric adenocarcinoma [54]. However, they showed the increased *DNMT1* gene expression was not correlated with the prognosis and clinicopathological including gastric adenocarcinoma area and grade tumor consonant with the present study (Table 6).

As the results of relevant other studies, the present study results showed that the *H. pylori cagA*, *cagE*, and *cagY* genotypes positively correlate with the *DNMT1* gene֝s expression [8]. The *H. pylori vacA* genotypes showed no statistical correlation with *DNMT1* gene֝s expression, which is in contrast with the results of previous studies [8]. In this case, the current study results support the correlation of antitumor VacA effects [55].

Although previous studies have shown the low prevalence of *H. pylori* and EBV co-infection in gastric epithelial cells, in the current study, no EBER was detected in gastric biopsy samples. So, the possible synergic role of EBV in increasing *DNMT1* genes֝ expression was waived.

*β*-Integrins are considered as CagY and CagT proteins֝ receptors that stimulate the cell signaling pathway toward cell growth and differentiation [56]. In the current study, the *cagY* genotype infection was statistically correlated with increased *DNMT1* gene֝ expression (Table 8).

## 5. Conclusion

This study's results indicate the correlation between *DNMT1* gene֝ expression in gastric antral epithelial cells and some gastric adenocarcinoma risk factors, including aging and *H. pylori* genotypes infection, which is consistent with the results of previous studies.

## Figures and Tables

**Table 1:** Primers of *H. pylori* T4SS, *16s rDNA*, and *vacA* in simple PCR.

Specific primers	Sequence	Annealing, Tm °C	Product size, bp	Reference
*16s rDNA H. pylori* F	CTGGAGAGACTAAGCCCTCC	50	446	This study
*16s rDNA H. pylori* R	AGGATCAAGGTTTAAGGATT			
*cagA* F	TGACCAACAACCACAAACCG	57	108	This study
*cagA* R	TCAGGATCGTATGAAGCGACAG			
*cagA* EPIYA-C F	AAGAAAGCAGGACAAGCAGC	55	188	This study
*cagA* EPIYA-C R	CTAACCGATCGCCCTACCTT			
*cagT* F	AGGGTGTGGTGATGATAGCG	55	154	This study
*cagT* R	TGCTTGTTGTTTGCTCCACT			
*cagE* F	GAATGGAGCGAGCGATGAAA	56	163	This study
*cagE* R	TAGGAATTTGCAGCGCTCAC			
*cagY* F	AGTTCAAGTGGCGCTAGATTG	57	200	This study
*cagY* R	ACAAGCCTTTCAAGCATTCGT			
*vacA* s1/s2 F	ATGGAAATACAACAAACACAC	55	259/286^*∗*^	[38]
*vacA* s1/s2 R	CTGCTTGAATGCGCCAAAC			
*vacA* m1/m2 F	TGGATAGTGCGACTGGGTTT	54	205/220^*∗*^	This study
*vacA* m1/m2 R	TCCATGCGGTTGTTGTTGTT			

^*∗*^The primers yield various *vacA* depending on a repetitive nucleotide sequence in some *vacA* alleles.

**Table 2:** Primers of *H. pylori* adhesins and surface proteins.

Specific primers	Sequence	Annealing, Tm °C	Product size, bp	Reference
*iceA1* F	GTGTTTTTAACCAAAGTATC	45	247	[39]
*iceA1* R	CTATAGCCASTYTCTTTGCA			
*iceA2* F	GTTGGGTATATCACAATTTAT	47	229/234	[39]
*iceA2* R	TTRCCCTATTTTCTAGTAGGT			
*hopQIe* F	ACGAACGCGCAAAAACTTTA	55	187	[40]
*hopQI* R	TTGCCATTCTCATCGGTGTA			
*hopQII* F	ACAGCCACTCCAATCCAGAA	55	160	[40]
*hopQII* R	AACCCCACCGTGGATTTTAG			
*babA2* F	CAATGCGGTGCGTGAAAATC	57	205	This study
*babA2* R	ATACCCTGGCTCGTTGTTGA			
*babB* F	CAATTCCCCGGCGTATCAAG	56	175	This study
*babB* R	ATTGCAAGTGATGGTCGTCG			
*sabA* F	TCTCTCGCTTGCGGTATCAT	56	204	This study
*sabA* R	AGCTCAATGTTGTTGGCGTT			
*sabB* F	GCATTCAAACGGCGAACAAC	56	248	This study
*sabB* R	TCCTGTGCAGTTCCCATCTT			
*alpA* F	CGCTCCTATCAAAACCGCTC	55	185	This study
*alpA* R	TTCCCGTCCAACTTACCGAA			
*alpB* F	TCAACTTGCGAGCAGACCTA	57	218	This study
*alpB* R	AGCCATAGACCCCATACACG			
*oipA* F	CTCCACGCTGAAAGGAATGG	55	233	This study
*oipA* R	CCATTTCCTGCGAATCGGTT			

**Table 3:** Specific primers of the *DNMT1* gene in real-time RT PCR.

Specific primers	Sequence	Annealing, Tm °C	Product size, bp	Reference
*GAPDH* F	ACCCTGTTGCTGTAGCCA	56	102	Universal
*GAPDH* R	CCACTCCTCCACCTTTGAC			
*DNMT1* F	AGAACGGTGCTCATGCTTACA	56	81	[41]
*DNMT1* R	CTCTACGGGCTTCACTTCTTG			
*EBER* F	AGGACAGCCGTTGCCCTAGTGGTT	60	173	This study
*EBER* R	AAAAATAGCGGACAAGCCGAATACC			

**Table 4:** Demographic characteristics of patients and correlation of demographic variables.

	Gastritis	Gastric adenocarcinoma	Total	*P* value ≤
*H. pylori* infection (positive/negative)	23/27	21/32	44/59	0.513
Sex (male/female)	24/26	37/16	50/53	0.024
Age group (18–30/31–45/46–60/61–85)	10/18/17/5	0/1/14/38	10/19/31/43	0.001

**Table 5:** Demographic characteristics of patients with *H. pylori* infection.

*H. pylori* infection (*n*: 103)	Sex (*n*)		Age group (*n*)			
	Male	Female	18–30	31–45	46–60	61–85
*H. pylori* infection	28	16	2	11	14	17
(Positive)						
*H. pylori* infection	33	26	8	8	17	26
(Negative)						
Total	61	42	10	19	31	43

**Table 6:** Correlation of *DNMT1* gene ΔCt in gastric antral epithelial cells of patients with gastritis and gastric adenocarcinoma with the Spearman statistical test patients' demographic characteristics.

	Sex	Age group	Disease	*H. pylori* infection	Gastric adenocarcinoma area	Tumor grade
DNMT1 correlation	−0.244	−0.475	−0.366	−0.472	−0.291	0.012
*P* value≤	0.094	0.001	0.010	0.001	0.189	0.956

**Table 7:** Frequency of *H. pylori* genotypes in gastric epithelial cells of patients with gastritis and gastric adenocarcinoma.

*H. pylori* ^*+*^ (*N*: 44)	Gastritis (*N*: 23)	Gastric adenocarcinoma (*N*: 21)	*P* value≤
*VacA s1m1*	7	12	0.074
*vacA s1m2*	16	9	
*cagA* ^*+*^	22	10	0.001
*cagA* ^*−*^	1	11	
*CagA*−*EPIYAC*^*−*^	22	10	0.001
*CagA*−*EPIYAC*^*+*^	1	9	
*CagT* ^*+*^	17	21	0.012
*CagT* ^*−*^	6	0	
*CagY* ^*+*^	5	17	0.001
*CagY* ^*−*^	18	4	
*CagE* ^*+*^	0	9	0.001
*CagE* ^*−*^	23	12	
*Sab* ^*+*^ *(A/B/AB)*	12/2/1	5/9/3	0.022
*Sab* ^*−*^	8	4	
*Bab* ^*+*^ *(A2/B/A2B)*	4/3/1	5/5/4	0.155
*Bab* ^*−*^	15	7	
*HopQ* ^*+*^ *(I/II)*	4/5	7/11	0.006
*HopQ* ^*−*^	14	3	
*Alp* ^*+*^ *(A/AB)*	11/6	11/7	0.610
*Alp* ^*−*^	6	3	
*OipA* ^*+*^	20	21	0.086
*OipA* ^*−*^	3	0	
*IceA2*	11	10	0.989
*iceA1/2*	12	11	

**Table 8:** Correlation between *DNMT1* gene ΔCt in gastric antral epithelial cells of gastritis and gastric adenocarcinoma patients with *H. pylori* virulence gene cDNAs using the Spearman test.

	*vacA s1m1/s1m2*	cagA	*cagA−EPEAYC*	*cagT*	*CagY*	*cagE*	*sabA/B*	*babA2/B*	*hopQI/II*	*alpA/B*	*oipA*	*iceA1/2*
DNMT1 correlation	0.116	−0.388	−0.293	0.209	−0.446	−0.517	0.313	0.176	0.155	−0.040	0.196	−0.366
*P* value ≤	0.589	0.023	0.093	0.326	0.029	0.002	0.136	0.411	0.469	0.853	0.359	0.078

**Table 9:** Analysis of the relationship between gastric diseases, tumor grade, *vacA* cDNA, and *H. pylori* infection variables with *DNMT1* gene ΔCt (*T*-test).

		*N*	Mean ΔCt *DNMT1*	Standard deviation	Fold change	*P* value≤
Age group	18–30	10	6.5789	1.72339	5.32	0.001
	61–85	43	4.1680	1.93520		

Disease	Gastritis	53	6.5227	1.60269	3.25	0.010
	Gastric adenocarcinoma	50	4.8226	1.89975		

*H. pylori* infection	Positive	44	4.7667	1.99491	3.11	0.001
	Negative	59	6.4035	1.49580		

*cagA*	Positive	16	6.1200	1.32237	3.04	0.023
	Negative	28	7.7267	1.66526		

*cagY*	Positive	23	5.9779	1.50656	3.08	0.029
	Negative	21	7.6030	1.62487		

*cagE*	Positive	15	6.1048	1.30111	4.09	0.002
	Negative	29	8.1360	1.48652		

## Data Availability

All data are already released in the article results, and the raw datasets used and/or analyzed during the current study are not publicly available due to contracts with research participants but are available from the corresponding authors upon reasonable request.

## References

[1] Zhang J., Yang C., Wu C., Cui W., Wang L. (2020). DNA methyltransferases in cancer: biology, paradox, aberrations, and targeted therapy. *Cancers*.

[2] Zeng X. Q., Wang J., Chen S. Y. (2017). Methylation modification in gastric cancer and approaches to targeted epigenetic therapy (Review). *International Journal of Oncology*.

[3] Etoh T., Kanai Y., Ushijima S. (2004). Increased DNA methyltransferase 1 (DNMT1) protein expression correlates significantly with poorer tumor differentiation and frequent DNA hypermethylation of multiple CpG islands in gastric cancers. *American Journal Of Pathology*.

[4] Jin B., Robertson K. D. (2013). DNA methyltransferases, DNA damage repair, and cancer. *Advances in Experimental Medicine and Biology*.

[5] Lin R. K., Wang Y. C. (2014). Dysregulated transcriptional and post-translational control of DNA methyltransferases in cancer. *Cell and Bioscience*.

[6] Denis H., Ndlovu M. N., Fuks F. (2011). Regulation of mammalian DNA methyltransferases: a route to new mechanisms. *EMBO Reports*.

[7] Benbrahim-Tallaa L., Waterland R. A., Dill A. L., Webber M. M., Waalkes M. P. (2007). Tumor suppressor gene inactivation during cadmium-induced malignant transformation of human prostate cells correlates with overexpression of de novo DNA methyltransferase. *Environmental Health Perspectives*.

[8] Sen P., Ganguly P., Ganguly N. (2018). Modulation of DNA methylation by human papillomavirus E6 and E7 oncoproteins in cervical cancer. *Oncology Letters*.

[9] Tolg C., Sabha N., Cortese R. (2011). Uropathogenic *E. coli* infection provokes epigenetic downregulation of CDKN2A (p16INK4A) in uroepithelial cells. *Laboratory Investigation*.

[10] Burgers W. A., Blanchon L., Pradhan S., de Launoit Y., Kouzarides T., Fuks F. (2007). Viral oncoproteins target the DNA methyltransferases. *Oncogene*.

[11] Chen C., Pan D., Deng A. M., Huang F., Sun B. L., Yang R. G. (2013). DNA methyltransferases 1 and 3B are required for hepatitis C virus infection in cell culture. *Virology*.

[12] Ksiaa F., Ziadi S., Gacem R. B., Dhiab M. B., Trimeche M. (2014). Correlation between DNA methyltransferases expression and Epstein-Barr virus, JC polyomavirus and *Helicobacter pylori* infections in gastric carcinomas. *Neoplasma*.

[13] Song J., Du Z., Ravasz M., Dong B., Wang Z., Ewing R. M. (2015). A protein interaction between *β*-catenin and Dnmt1 regulates wnt signaling and DNA methylation in colorectal cancer cells. *Molecular Cancer Research*.

[14] Campbell P. M., Szyf M. (2003). Human DNA methyltransferase gene DNMT1 is regulated by the APC pathway. *Carcinogenesis*.

[15] Guo Y., Wang M., Jia X., Zhu H., Zhi Y., Yuan L. (2018). Wnt signaling pathway upregulates DNMT1 to trigger NHERF1 promoter hypermethylation in colon cancer. *Oncology Reports*.

[16] Song X., Xin N., Wang W., Zhao C. (2015). Wnt/*β*-catenin, an oncogenic pathway targeted by *H. pylori* in gastric carcinogenesis. *Oncotarget*.

[17] Pacis A., Tailleux L., Morin A. M. (2015). Bacterial infection remodels the DNA methylation landscape of human dendritic cells. *Genome Research*.

[18] Silva-García O., Valdez-Alarcón J. J., Baizabal-Aguirre V. M. (2019). Wnt/*β*-Catenin signaling as a molecular target by pathogenic bacteria. *Frontiers in Immunology*.

[19] Fan H., Zhao Z., Quan Y., Xu J., Zhang J., Xie W. (2007). DNA methyltransferase 1 knockdown induces silenced CDH1 gene reexpression by demethylation of methylated CpG in hepatocellular carcinoma cell line SMMC-7721. *European Journal of Gastroenterology and Hepatology*.

[20] Zebardast S., Sahmani M., Mohammadi S. (2020). The gene expression profile and DNA methylation pattern of CDH1 and DNMT1 genes in acute promyelocytic leukemia (APL). *Reports of Biochemistry and Molecular Biology*.

[21] Ma F., Lei Y. Y., Ding M. G., Luo L. H., Xie Y. C., Liu X. L. (2020). LncRNA NEAT1 interacted with DNMT1 to regulate malignant phenotype of cancer cell and cytotoxic T cell infiltration via epigenetic inhibition of p53, cGAS, and STING in lung cancer. *Frontiers in Genetics*.

[22] Zhang B. G., Hu L., Zang M. D. (2016). *Helicobacter pylori* CagA induces tumor suppressor gene hypermethylation by upregulating DNMT1 via AKT-NF*κ*B pathway in gastric cancer development. *Oncotarget*.

[23] Muhammad J. S., Eladl M. A., Khoder G. (2019). *Helicobacter pylori*-Induced DNA methylation as an epigenetic modulator of gastric cancer: recent outcomes and future direction. *Pathogens*.

[24] Silva-García O., Valdez-Alarcón J. J., Baizabal-Aguirre V. . M. (2014). The wnt/*β*-catenin signaling pathway controls the inflammatory response in infections caused by pathogenic bacteria. *Mediators of Inflammation*.

[25] Smatti M. K., Al-Sadeq D. W., Ali N. H., Pintus G., Abou-Saleh H., Nasrallah G. K. (2018). Epstein-barr virus epidemiology, serology, and genetic variability of LMP-1 Oncogene among healthy population: an update. *Frontiers in Oncology*.

[26] Okabe A., Funata S., Matsusaka K. (2017). Regulation of tumour related genes by dynamic epigenetic alteration at enhancer regions in gastric epithelial cells infected by Epstein-Barr virus. *Scientific Reports*.

[27] Pandey S., Jha H. C., Shukla S. K., Shirley M. K., Robertson E. S. (2018). Epigenetic regulation of tumor suppressors by *Helicobacter pylori* enhances EBV-induced proliferation of gastric epithelial cells. *mBio*.

[28] Matsusaka K., Funata S., Fukayama M., Kaneda A. (2014). DNA methylation in gastric cancer, related to *Helicobacter pylori* and epstein-barr virus. *World Journal of Gastroenterology*.

[29] Szkaradkiewicz A., Karpiński T. M., Majewski J., Malinowska K., Goslinska-Kuzniarek O., Linke K. (2015). The participation of p53 and bcl-2 proteins in gastric carcinomas associated with *Helicobacter pylori* and/or epstein-barr virus (EBV). *Polish Journal of Microbiology*.

[30] Cárdenas-Mondragón M. G., Carreón-Talavera R., Camorlinga-Ponce M., Gomez-Delgado A., Torres J., Fuentes-Panana E. M. (2013). Epstein barr virus and *Helicobacter pylori* Co-infection are positively associated with severe gastritis in pediatric patients. *PLoS One*.

[31] Cao X. Y., Ma H. X., Shang Y. H. (2014). DNA methyltransferase3a expression is an independent poor prognostic indicator in gastric cancer. *World Journal of Gastroenterology*.

[32] Carlson R. V., Boyd K. M., Webb D. J. (2004). The revision of the Declaration of Helsinki: past, present and future. *British Journal of Clinical Pharmacology*.

[33] Lankarani K., Moosazadeh M., Afshari M. (2016). Meta-analysis of the prevalence of *Helicobacter pylori* infection among children and adults of Iran. *International Journal of Preventive Medicine*.

[34] Charan J., Biswas T. (2013). How to calculate sample size for different study designs in medical research?. *Indian Journal of Psychological Medicine*.

[35] Nayak B. K., Hazra A. (2011). How to choose the right statistical test?. *Indian Journal of Ophthalmology*.

[36] Telloni S. M. (2017). Tumor staging and grading: a primer. *Methods in Molecular Biology*.

[37] Lorenz T. C. (2012). Polymerase chain reaction: basic protocol plus troubleshooting and optimization strategies. *Journal of Visualized Experiments: Journal of Visualized Experiments*.

[38] Atherton J. C., Cao P., Peek R. M., Tummuru M. K., Blaser M. J., Cover T. L. (1995). Mosaicism in vacuolating cytotoxin alleles of *Helicobacter pylori*. *Journal of Biological Chemistry*.

[39] van Doorn L., Figueiredo C., Sanna R. (1998). Clinical relevance of the cagA, vacA, and iceA status of *Helicobacter pylori*. *Gastroenterology*.

[40] Sicinschi L. A., Correa P., Bravo L. E. (2012). Non-invasive genotyping of *Helicobacter pylori* cagA, vacA, and hopQ from asymptomatic children. *Helicobacter*.

[41] Xu H., Chen Y., Chen Q. (2017). DNMT1 regulates IL-6- and TGF-*β*1-induced epithelial mesenchymal transition in prostate epithelial cells. *European Journal of Histochemistry*.

[42] Kralik P., Ricchi M. (2017). A basic guide to real time PCR in microbial diagnostics: definitions, parameters, and everything. *Frontiers in Microbiology*.

[43] Cover T. L. (2016). *Helicobacter pylori* diversity and gastric cancer risk. *mBio*.

[44] Herrera V., Parsonnet J. (2009). *Helicobacter pylori* and gastric adenocarcinoma. *Clinical Microbiology and Infections*.

[45] Kumar S., Metz D. C., Ellenberg S., Kaplan D. E., Goldberg D. S. (2020). Risk factors and incidence of gastric cancer after detection of *Helicobacter pylori* infection: a large cohort study. *Gastroenterology*.

[46] Ksiaa F., Ziadi S., Dhiab M. B., Gacem R. B., Trimeche M. (2015). Increased DNA methyltransferase 1 protein expression correlates significantly with intestinal histological type and gender in gastric carcinomas. *Advances in Medical Sciences*.

[47] Liang Y. Y., Lin J. H. (2015). Preliminary research of PDCD4 and DNMT1 expression in human gastric cancer tissues. *Experimental and Laboratory Medicine*.

[48] Sun N., Lu X. F., Liu S. N., Zheng X. T., Li Q. Q. (2011). Expression and significance of PDCD4 and DNMT1 in gastric carcinoma. *Medicine and Philosophy (Clinical Decision Making Forum Edition)*.

[49] Liu B., Gu L. P., Xing C. P. (2009). Relationship between expression of Dnmt1 and caveolin-1 in gastric cancer. *Journal of the Fourth Military Medical University*.

[50] Liu T., Cao M. Q., Li X. Y., Yang W. L. (2007). Expression of DNA methyltransferase 1 in human gastric cacner tissue and its significancer. *Shanghai Medical Journal*.

[51] Ciccarone F., Malavolta M., Calabrese R. (2016). Age-dependent expression of DNMT1 and DNMT3B in PBMCs from a large European population enrolled in the MARK-AGE study. *Aging Cell*.

[52] Yong X., Tang B., Li B. S. (2015). *Helicobacter pylori* virulence factor CagA promotes tumorigenesis of gastric cancer via multiple signaling pathways. *Cell Communication and Signaling*.

[53] Nakayama M., Hisatsune J., Yamasaki E. (2009). *Helicobacter pylori* VacA-induced inhibition of GSK3 through the PI3K/Akt signaling pathway. *Journal of Biological Chemistry*.

[54] Ma T., Li H., Sun M., Yuan Y., Sun L. P. (2017). DNMT1 overexpression predicting gastric carcinogenesis, subsequent progression and prognosis: a meta and bioinformatic analysis. *Oncotarget*.

[55] Rassow J. (2011). *Helicobacter pylori* vacuolating toxin A and apoptosis. *Cell Communication and Signaling*.

[56] Zhao Q., Busch B., Jiménez-Soto L. F. (2018). Integrin but not CEACAM receptors are dispensable for *Helicobacter pylori* CagA translocation. *PLoS Pathogens*.

